# Preparation of a Hybrid Membrane from Whey Protein Fibrils and Activated Carbon to Remove Mercury and Chromium from Water

**DOI:** 10.3390/membranes10120386

**Published:** 2020-11-30

**Authors:** Laura Cristina Ramírez-Rodríguez, Luis Eduardo Díaz Barrera, María Ximena Quintanilla-Carvajal, Didilia Ileana Mendoza-Castillo, Adrián Bonilla-Petriciolet, Carlos Jiménez-Junca

**Affiliations:** 1 Maestría en Diseño y Gestión de Procesos Facultad de Ingeniería, Campus Universitario Puente del Común, Universidad de la Sabana, Km. 7 Autopista Norte, 140013 Chía, Colombia; lauraramro@unisabana.edu.co; 2 Bioprospecting Research Group, Campus Universitario Puente del Común, Universidad de La Sabana, Km. 7 Autopista Norte, 140013 Chía, Colombia; luis.diaz1@unisabana.edu.co; 3 Agroindustrial Processes Research Group, Campus Universitario Puente del Común, Universidad de La Sabana, Km. 7 Autopista Norte, 140013 Chía, Colombia; maria.quintanilla1@unisabana.edu.co; 4 Cátedras CONACYT, Instituto Tecnológico de Aguascalientes, Avenida Adolfo López Mateos #1801, Aguascalientes 20256, Mexico; didi_men@hotmail.com; 5 Departamento de Ingeniería Química, Instituto Tecnológico de Aguascalientes, Aguascalientes 20256, Mexico; petriciolet@hotmail.com

**Keywords:** activated carbon, heavy metals, membrane, protein water treatment, whey

## Abstract

Water contamination by mercury and chromium has a direct effect in human health. A promising technology to remove heavy metals by membrane filtration is the use of hybrid membranes produced with whey protein fibrils (WPF) and activated carbon (AC). In this study, the best conditions to produce WPF by heat treatment were determined to maximize the removal of mercury and chromium from water using a central composed design. The results indicated that the best conditions to prepare WPF were 74 °C, 7 h and 3.8% of whey protein with adsorption capacities of 25 and 18 mg/g and removal efficiencies of 81 and 57% for mercury and chromium, respectively. WPF and AC were used to prepare a hybrid membrane that was characterized using transmission electron microscopy, atomic force microscopy, scanning electron microscopy, Fourier transform infrared spectroscopy and Brunauer–Emmett–Teller surface area measurements. Batch filtration experiments were performed with the hybrid membrane for chromium and mercury removal at 25, 50 and 100 mg/L to determine its adsorption capacities. A high performance of the hybrid membrane was demonstrated removing efficiently mercury and chromium from water, thus supporting more than ten filtration cycles.

## 1. Introduction

Water pollution affects millions of people around the world, which leads to health problems and can cause death in populations under extreme conditions [1]. The major sources of water pollution by toxic ions are unregulated industries, energy production and mining, which have substantially increased the pollutants concentration in water [2]. Heavy metal ions are not biodegradable and have a massive effect in the ecosystem. As a result, their bioaccumulation and biomagnification are present in organisms and the food chain [3]. Therefore, heavy metals cause serious health risks and environmental impacts [4].

Chromium and mercury are recognized as a potential risk to human health and the environment due to their carcinogenic, toxic and mutagenic nature. Chromium, especially its hexavalent state (Cr (VI)), is toxic, soluble in water, has high mobility and can induce autophagy of human stem cells [5]. This metal is used in steel industries, pigments manufacture, the electroplating industry and as a leather tanning agent [6]. Likewise, mercury is considered the most toxic heavy metal in the environment and is one of the priority pollutants according to the US EPA [7]. Owing to the transformation of mercury by microorganisms into methylmercury, it is bio-magnified in the environment causing adverse effects in organisms. The main sources of mercury pollution are agriculture, urban wastewater, mining, incineration and industrial wastewater [8].

To remove heavy metal ions from water, various physical and chemical methods have been used, such as chemical precipitation [4], flocculation, coagulation [9], ion-exchange [10] and electrochemical treatment techniques [11]. However, the implementation of these techniques faces economic or environmental constraints owing to the high cost, demanding additional processes to eliminate waste products, specific operating conditions to ensure an acceptable decontamination, and some of these techniques face the problem that they could be effective to remove only one specific heavy metal ion [6]. Among these methods for heavy metal removal, adsorption is an efficient technology because of its simple operation, low operating cost (especially, energy), low sludge production and the recyclability of the adsorbent to remove metal ions from dilute and concentrated effluents [12]. However, adsorption has some disadvantages, such as the high cost of commercial adsorbents and a difficult implementation with respect to the removal of complex ions [13]. Consequently, a promising and emerging technology is the production of adsorbent membranes due to it combining the adsorption properties of the material and the filtration performance of the membrane [14,15]. Research efforts have focused on the use of hybrid materials such as protein amyloids and activated carbon for wastewater treatment, which include the development of new types of membranes by self-assembly of adsorbents [16,17,18].

In this way Bolisetty and Mezzenga [16] produced amyloid fibrils by heat treatment using β-lactoglobulin to obtain a hybrid membrane with activated carbon, which was able to remove more than 99.5% of gold, mercury, lead and palladium, individually and mixed, in aqueous solution. Using the same type of membrane, 99% of arsenites and arsenates were removed from prepared solutions and contaminated real water [17]. To comprehend the mechanisms of the metal ion binding with β-lactoglobulin amyloids, Peydayesh et al. [18] studied the adsorption isotherms using isothermal thermal calorimetry (ITC) on native β-lactoglobulin monomers. This study was performed on the removal of chromium (Cr), nickel (Ni), silver (Ag) and platinum (Pt), where the results showed removal efficiencies beyond 99%.

Similarly, Mezzenga et al. [19] reported the use of hybrid membranes made of β-lactoglobulin amyloid fibrils-ZrO2 nanoparticles (<10 nm) that efficiently removed fluoride from polluted water. These hybrid membranes presented a high selectivity for fluoride against various competitive ions and exceeded the performance of most commercial carbon-activated aluminum membranes. The efficiency of the membrane exceeded 99.5% removal.

Additionally, Yu et al. [2] produced a hybrid membrane for the detection and separation of mercury ion, which was produced by filtrating gold nanocluster with bovine serum albumin nanofibers and graphene oxide. These membranes presented a removal efficiency of mercury up to 90.4% in the first cycle. Furthermore, Yu et al. [2] reported the preparation of a graphene oxide (GO)-bovine serum albumin (BSA) hybrid membrane for metal ions detection, which has a large surface area and a high metal-binding ability due to BSA.

These studies demonstrated interesting results for heavy metal removal from water using these types of hybrid membranes. The high performance of the membranes is due to the synergy of high metal binding capacity of proteins with a high surface area material [18]. From these works, it can be concluded that most of the hybrid membranes involve β-lactoglobulin or BSA alone as a protein source, which opens the opportunity to produce hybrid membranes from a mix of proteins like whey.

Whey is a by-product of the cheese industry composed mainly by β-lactoglobulin (60%) and α-lactalbumin (20%), which causes a pollution problem especially in countries connected to the milk economy because nine kilograms of whey are generated per each kilogram of cheese [20,21]. Yet, in nations such as U.S., Australia and China, whey manufacture and consumption are growing mainly due to infants and sport nutrition, with an annual growth rate of 8.1% [22] and an average price of 6 USD/Kg for 2018 [21]. Despite this, some companies have tried to enter the market of protein isolation by fractionation but lacked success due to the added value in the production price of isolated proteins, such as β-lactoglobulin, which costs 5.43 USD per kilogram of treated whey protein isolate (WPI) [23] and also includes a cumbersome and time-consuming additional process [24,25]. For that reason, an application at large scale using a hybrid adsorbent of β-lactoglobulin, as proposed in a number of studies [16,17,18], is not viable due to the high cost of the protein purification [26].

Consequently, the use of whey is an opportunity to make hybrid materials that have many advantages owing to the combination of the properties of the components and strong economic incentives. Thus, in this study, whey protein fibrils (WPF) were used to prepare a hybrid membrane with activated carbon (AC) because it is a well-known adsorption material owing to its large surface area and can provide mechanical support to WPF. Additionally, the use of hybrid materials may lead to a lower-cost material than activated carbon (10 USD/Kg) [27,28] alone because of the combination of a low-cost feedstock, as whey, with activated carbon. This novel hybrid membrane was tested for mercury and chromium removal from water. This work considered it important to find the optimal conditions to produce WPF from whey to remove heavy metals before preparing the hybrid membrane, which has not been reported in the literature. The results demonstrated that the production conditions of WPF affected the way proteins aggregate so that it can lead to a significant impact on the removal of mercury and chromium from water. Furthermore, this hybrid membrane showed promising performance in removing heavy metals after various filtering cycles.

## 2. Materials and Methods

### 2.1. Materials

Whey protein isolate (WPI) was purchased from Davisco Foods International Inc. (Eden Prairie, MN, USA) and it was 97% protein of which approximately 69% was β-Lactoglobulin and 22% was α-lactalbumin. β-Lactoglobulin (>90%), mercury chloride (II), sodium chromate and activated carbon (particle size of 45 µm) were obtained from Sigma Aldrich (St. Louis, MO, USA). All reagents were analytical grade and used as received. Nitrocellulose membranes (Sartorius Stedim Biotech, Goettingen, Germany) with a pore size of 0.22 µm were used to produce the hybrid membrane.

### 2.2. Preparation of Protein Fibrils

Amyloids were prepared following the method proposed by Peydayesh et al. [18]. Solutions were prepared according to a central composited design (CCD, see Table 1), which was used to evaluate the impact of whey concentration, temperature and time on the adsorption capacity of heavy metals. This CCD included 15 trials with five levels for each factor. In particular, whey powder was dissolved in deionized water at concentrations of 0.5–5 wt%. β-Lactoglobulin was prepared as reference at the conditions obtained by CCD and all samples were adjusted at pH 2 because this condition ensured fibril formation due to the electrostatic repulsion among positively-charged groups, monomers and peptides preventing a random aggregation [29]. The solution was centrifuged at 10,800 RFC for 1 h at 20 °C using a centrifuge (Universal 32R, Tuttlingen, Germany), and filtered through a 0.45 µm Millipore filter before a heat-treatment to remove any traces of undissolved protein. A volume of 15 mL of solution were placed in glass tubes hermetically sealed and maintained in a water bath without stirring at 65–90 °C for 5–11 h. After this treatment, the tubes were immediately cooled by immersion in ice-water mixtures, and also a sample that was not heated was used as reference [30].

The experimental results of CCD were statistically studied by an analysis of variance (ANOVA). Design-Expert 10.0 software (Stat-Ease, US) was used to analyse the experimental design, response surface modelling, statistical regression analysis and process optimization. The results were fitted via the response surface regression procedure using a second order polynomial given by Equation (1) [31]:

(1) $$Y=\beta_{0}+\overset{n}{\underset{i=1}{\sum }}\beta_{i}x_{i}+\overset{n-1}{\underset{i=1}{\sum }}\overset{n}{\underset{j=i+1}{\sum }}\beta_{ij}x_{i}x_{j}+\overset{n}{\underset{i=1}{\sum }}\beta_{ii}x_{i}{}^{2}$$

where *Y* is the predicted response, *β*__0__ is the regression coefficients, *β_i_* is the linear coefficient, *β_ii_* is the quadratic coefficients, *β_ij_* is the interaction coefficients and *X_i_* is the coded levels of independent variables, respectively.

### 2.3. Preparation of Hybrid Membranes

Hybrid nanomembranes were prepared using the method of Li et al. [32]. A whey fibril solution produced at the best conditions at pH 2 was dropped into the AC solution (0.1 wt%) with vigorous stirring to give a final mixture of 1:0, 2:1, 1:1, 1:2, 1:5 and 0:1 of AC–WPF. The protein hybrid membranes were prepared by vacuum filtration of 1.8 mL of the mixture solution, passing the mixture through a nitrocellulose filter of 0.22 μm and diameter of 47 mm.

### 2.4. Characterization of Protein Fibrils and Hybrid Membranes

Fibril formation was analysed by transmission electron microscopy (Quantifoil, Großlöbichau, Germany) and atomic force microscopy (Asylum Research, Goleta, CA, USA). AFM samples were held in cleaved mica sheets and rinsed with Milli-Q water. The size distributions and shapes of fibrils were measured using a cantilever operated in tapping mode for image topography. The cantilever was silicon nitride with a nominal force constant of 0.08 N/m, the scan rate was set to 1 Hz [33] and Gwyddion^®^ v. 2.54 (Czech Metrology Institute, Brno, Czech republic) free software was used for image processing. The morphology of hybrid membrane was analysed with a Tescan LYRA 3 scanning electron microscope (SEM) (TESCAN, Brno, Czech Republic) at an acceleration voltage of 3 kV [2].

The amyloid fibril formation was determined by Congo Red assay following the method proposed by Yakupova et al. [34]. A qualitative chemical characterization of fibrils, activated carbon and the hybrid composite at different compositions 1:0, 1:1, 1:2, 1:5 and 0:1 was performed by Fourier transform infrared spectroscopy (FTIR) (Thermo Scientific^®^, Waltham, MA, USA) in the region of 4000–500 cm^−1^ [19]. Surface area was determined using Brunauer–Emmett–Teller (BET) method and measured by nitrogen adsorption at 77 K (i.e., −196 °C) in a ChemBET Pulsar TPR/TPD unit (Quantachrome instruments, Boynton Beach, FL, USA) [35].

### 2.5. Removal Efficiency of Heavy Metals from Water

Batch experiments and filtration were carried out to evaluate the adsorption capacities of heavy metals on WPF and hybrid membranes. Prior to the experiment, stock aqueous solutions of mercury chloride (II) and sodium chromate of different initial concentrations were adjusted to pH 3.0 using 0.1 M of HNO_3_. Note that Cr and Hg exist as dissolved anion (HCrO_4_^2−^) [36] and cation (Hg^2+^) [37], respectively, in the aqueous solution at the tested pH according to speciation diagrams. To determinate the removal efficiency of WPF, the solution obtained by heat treatment with a known quantity of fibrils was equilibrated (1:1 wt) with a heavy metal solution of 100 mg/L to have a final concentration of 50 mg/L. The experiment was carried out under isothermal conditions at room temperature (20 °C), pH 3 and continuous shaking (150 rpm) for 24 h to ensure the equilibrium. Finally, the resulting solution was centrifuged, filtered and analysed with a ContrAA 700 atomic absorption spectrophotometer (Analytik Jena, Jena, Germany). Blank experiments were also performed to ensure that no adsorption took place on the walls of the apparatus used.

To evaluate the removal performance of hybrid membranes, 50 mL of a heavy metal solution with a given initial concentration (i.e., 25, 50, 100 mg/L) was filtered through WPF-AC hybrid membrane (note that a new membrane was available for each heavy metal concentration tested) and the metal concentration was measured before and after filtration by a ContrAA 700 atomic absorption spectrophotometer (Analytik Jena, Jena, Germany). The removal capacity was determined by filtering consecutive cycles of 50 mL of heavy metal solutions and the residual concentration of the metals in the filtrate was measured until a decrease in the removal efficiency, compared to the initial one, was observed. The removal of heavy metals was determined using only activated carbon and only whey protein fibrils, and these values were used as a reference [17]. The removal efficiency (R, %) and adsorption capacity (q, mg/g) [28] were calculated with the following equations:

(2) $$R\left(\%\right)=\frac{C_{0}-C_{t}}{C_{0}}\times 100$$

(3) $$q=\frac{\left(C_{0}-C_{t}\right)}{m_{s}}\times V$$

where *C*_0_ is the initial heavy metal concentration, *C_t_* is the heavy metal concentration after filtration, *V* is the heavy metal solution volume (L) and m_s_ is the weight of hybrid membrane (g), respectively.

### 2.6. Statistical Analysis

Analysis of variance (ANOVA) was conducted with CDD results followed by LSD test to establish statistical differences between treatments with a significance level (*p*-level) of 0.05. Experiments were performed in triplicate, and the results were reported as the mean and standard deviation of the measurements. SPSS V24 software was employed in data processing.

## 3. Results

### 3.1. Preparation of Whey Protein Fibrils

Results of CCD were used to determinate the best conditions to prepare WPF. The experimental data of the adsorption capacity and efficiency of WPF to remove mercury and chromium are shown in Table 2. ANOVA indicated that all tested parameters of WPF production had a significant effect (*p* < 0.05) on the adsorption capacity and efficiency for mercury, while the quadratic term associated to the temperature in the model given by Equation (1) was not significant. The same result was found for the removal efficiency of chromium, but the interaction term between temperature and protein was not significant in this model. In the literature, many researchers have concluded that the WPF aggregation and conversion by heat treatment depended of temperature, protein content and time. Therefore, the way that these factors interfere on the fibril formation may influence the adsorption of heavy metals on fibrils [38,39,40,41]. The conditions that favor the fibrils production found in the literature were used as starting point to design the experiment and to obtain whey protein fibrils.

A two-factor interaction and a quadratic model were used to analyze the interaction between the independent factors and the effect on adsorption capacity and removal efficiency of mercury and chromium. These models are given in Equations (4) and (5) for mercury and Equations (6) and (7) for chromium was used. R^2^ was higher than 0.99 and the adjusted R^2^ was higher than 0.98 for all models where lack of fit was 0.694 and 0.227 (nonsignificant) for mercury and chromium, respectively.

These models for the calculation of the adsorption capacity q (mg/g) at 50 mg/L and removal efficiency (%) for mercury and chromium were defined as follows, Equations (4)–(7):

(4) $$\begin{array}{c} q\;Hg\;\left(mg/g\right)=250.02-3.59*A-38.88*B+63.34*C+0.54*A*B-0.49*A*C\\ \;\;\;\;\;\;-1.01*B*C)+0.0019*A^{2}-0.22*B^{2}-2.32*C^{2} \end{array}$$

(5) $$\begin{array}{c} Removal\;Efficiency\;Hg\;\left(\%\right)\\ \;\;\;\;\;\;=848.59-12.21*A-131.96*B+214.98*C+1.83*A*B-1.67*A\\ {}*C-3.43*B*C+0.0064*A^{2}-0.75*B^{2}-7.89*C^{2} \end{array}$$

(6) $$q\;Cr\;\left(mg/g\right)=240.47-4.09*A-16.22*B+11.64*C+0.22*A*B+0.0306*A*C\;-1.44*B*C+0.0104*A^{2}+0.066*B^{2}+0.17*C^{2}\;\;\;$$

(7) $$\begin{array}{c} Removal\;Efficiency\;Cr\;\left(\%\right)\\ \;\;\;\;\;\;\;\;=762.68-12.97*A-51.44*B+36.93*C+0.72*A*B+0.097*A*C\\ -4.58*B*C+0.03*A^{2}+0.21*B^{2}+0.54*C^{2}\;\;\;\; \end{array}$$

where *A* is temperature (°C), *B* is time (h) and *C* is whey concentration (wt%).

The results obtained from CCD indicated that the temperature, protein content and time had a significant effect on mercury and chromium adsorption capacity and removal efficiency. Consequently, it confirmed that these factors have a critical effect on whey proteins aggregation [39,42], which has a relationship with the mechanism to remove heavy metals from water because, depending on the way amyloid fibrils arranged, the active sites of the fibrils will be more or less available [43]. Furthermore, the models obtained in this study (Equations (4)–(7)) indicated that protein content had a positive influence on Hg and Cr removal capacity and efficiency, while temperature and time showed a negative effect on the removal of Hg and Cr. For instance, there must be an equilibrium between the protein content and the heating conditions to prepare WPF. Whey solutions with high protein content submitted to high temperatures for a long heating time, instead of producing active amyloids fibrils to remove heavy metals, generate protein gels with low removal capacity [30]. As shown in Table 3, the best conditions to produce WPF and to improve the adsorption capacity and efficiency for mercury and chromium removal were: a temperature near to 74 °C, at approximately 7 h with a protein concentration of 3.8%. So that, the preparation conditions of WPF to remove both mercury and chromium were very similar, showing that the aggregation conditions affect the adsorption capacity and removal efficiency of these heavy metals in the same way. Also, non-heated whey protein solutions were used as reference and they did not remove Hg or Cr.

However, these results also showed that WPF had a better affinity to mercury than chromium, which is due to the chemical interactions and the intermolecular forces between the functional groups of WPF with heavy metals [44]. In this study, the adsorption capacity and removal efficiency of WPF for Hg and Cr were evaluated at pH 3, which is a critical operating parameter to determine the interaction between metal ions and WPF due the solution pH can change the physicochemical properties of WPF and metal ions. The surface charge of WPF is affected by the pH and also it influences the way as the metal ion is presented in the solution, as Hg that is presented as a free cation and Cr is presented as a complex anion (HCrO_4_^2−^) at pH 3 [45]. Taking into consideration that the isoelectric point of β-Lactoglobulin amyloid fibrils is approximately 5.1 [46], the surface charge of WPF was positively charged at the evaluated conditions leading an electrostatic interaction between HCrO_4_^2−^ and WPF. On the other hand, Hg^2+^ is classified as a soft ion according to the HSAB theory, which means that it forms very strong bonds with –SH, –SR, –CN, –NH_2_ and imidazole groups containing nitrogen and sulfur atoms [47]. As consequence, WPF are composed by amide group and cysteines that have a high affinity for Hg. A high presence of sulfur bonds due to the cysteine of whey proteins, such as the four disulfide bonds of α-lactalbumin (Cys6–Cys120, Cys61–Cys77, Cys73–Cys91, and Cys28–Cys111) [48] and the sulfur of the Cys121 of β-Lactoglobulin, make WPF promising for Hg removal. Various researchers have confirmed that the Cys121 of β-Lactoglobulin at low pH has high affinity for cations such as Hg^2+^ and Cd^2+^ [49].

The model used in this study to obtain the best conditions to produce WPF predicted a 100% for the removal of Hg, but a maximum removal efficiency of 81% was obtained experimentally using an initial Hg concentration of 50 mg/L. This was due to the preparation conditions affected the way as whey protein aggregated into WPF because temperature and heating time impacted the degree of unfolding and, in consequence, the available sites and surface energy to adsorb metal ions [42]. As β-lactoglobulin unfolds to form amyloid fibrils, several peptides participate in fibril formation, and cys-121 is one of them [16]. So that, there are adsorption limits that the model at tested conditions does not consider and there was an error of 19% between predicted and experimental values of the removal efficiency [50]. It can be concluded that WPF is a promissory material to remove mercury considering its removal efficiency was 81%, which compared with β-Lactoglobulin that had a removal efficiency of 90%, the cost-effective performance is outstanding.

The results indicated that the best operational conditions produced WPF to remove the maximum quantity of mercury and chromium alone and mixed. Similar conditions (i.e., 75~120 °C, pH 2~3.5, low salt content < 0.1 M and 60~85 °C, pH 2) are commonly used to form amyloid fibrils of β-Lactoglobulin [29,41,51,52] and α-lactalbumin [48,53,54], respectively. Loveday et al. [55] concluded that a protein sequence exposed to different conditions can generate a variety of a fibrillar aggregations. These findings are in agreement with this work even when there is a variety of proteins interacting on whey protein, they can form active aggregates at the adequate conditions to remove heavy metals. In this study, this type of aggregates, named as WPF, presented an adsorption capacity of 24 and 18 mg/g for chromium and mercury removal using 50 mg/L of initial concentration, respectively, see Table 3. Then, an adsorption capacity of 27 and 19 mg/g was obtained for Hg and Cr using β-lactoglobulin fibrils as reference. There was not a significant difference between the adsorption capacity obtained for chromium using WPF and β-lactoglobulin fibrils, while the adsorption capacity to remove mercury using WPF and β-lactoglobulin only differed by 8% making this type of aggregates of whey promissory to remove toxic metal ions. Similarly, Zhang et al. [56] produced β-lactoglobulin nanofibrils at 90 °C, 5% *w*/*v*, pH 12 for 10 h, which were used to remove Pb with a maximum adsorption capacity of 212 mg/g. This result demonstrates the high potential of protein nanofibers to remove heavy metals from water.

Adsorption capacities of 24.13 and 11.55 mg/g for mercury and chromium in a mixed solution (see Table 3) confirmed that WPF had more affinity for mercury. Note that mercury has a strong affinity to the functional groups of (–NH_2_) [57] and (–COOH) of the R group of amino acids, causing that the groups alpha amino and carboxyl are involved in the peptide bonds of the proteins and cannot interact with metal ions [58]. Additionally, the presence of cysteine and methionine in whey proteins promotes affinity from WPF to mercury though deprotonated thiol group [59], and also the deprotonation of tyrosine may generate a ligand to Hg [44]. However, the results showed that chromium was adsorbed also in the mixed solution. Thus, it means that functional groups of proteins like amine [60] and hydroxyl groups [61] favor the chromium adsorption, which is well known that these functional groups have an electrostatic effect on chromium adsorption. It can be concluded that WPF show specificity for mercury due to the interactions between the functional groups of whey proteins and metal ion Hg [5,62].

### 3.2. Characterization of Protein Fibrils and Hybrid Membranes

#### 3.2.1. AFM Characterization of Whey Protein Aggregates

**Figure 1:**
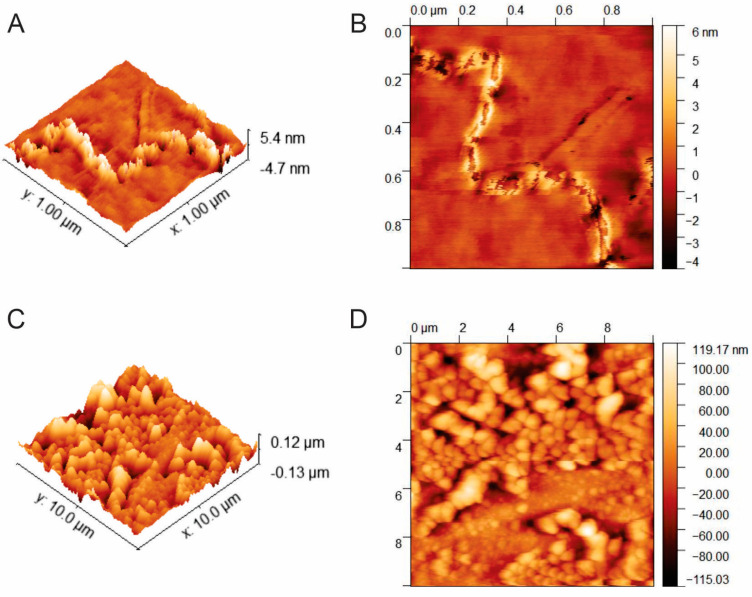
Morphological analysis of whey fibrils compared with β-lactoglobulin fibrils by AFM. (**A**) 3D reconstruction of AFM image of β-lactoglobulin fibrils; (**B**) image of morphology of β-lactoglobulin fibrils; (**C**) 3D reconstruction of AFM image of whey protein aggregates; (**D**) image of morphology of whey protein aggregates.

AFM characterization showed the transformation of native β-lactoglobulin and whey protein into aggregates at the best conditions to remove heavy metals (3.8% wt, 74 °C for 7 h) (see Figure 1). In particular, Figure 1A,B shows the morphology and height distribution of β-lactoglobulin as 5 nm, which was similar with the height between 4–10 nm reported in the literature [30,40,63,64]. The difference of diameter in fibrils varies due to the denaturation conditions of the proteins, such as monomer concentration, temperature and incubation time, which led to protofilaments that form twisted fibrils from one protofilament until three filaments twisted [43].

On the other hand, Figure 1C,D shows the aggregation of native whey at the best conditions to remove Hg and Cr. The height of aggregates was 13 nm, which was in agreement with the diameter of 5–15 nm reported in the literature for whey fibrils aggregates [65,66,67]. In addition, the height of whey protein aggregates is higher than β-lactoglobulin aggregates because whey protein aggregates at smaller rate than β-lactoglobulin due to α-lactalbumin aggregates slower when heated than β-lactoglobulin [65]. Many authors confirmed the conversion of whey proteins onto fibrils aggregates at similar conditions evaluated in this work because binary mixtures of proteins with β-lactoglobulin tend to arrange as fibrils at the suitable denaturation conditions. For example, Bolder et al. [39] confirmed the aggregation of whey onto long semi-flexible fibrils by TEM. A recent study by Farrokhi et al. [66] demonstrated the fibrillation of whey by AFM via the heating of a solution of 2% of WPI at 80 °C and pH 2. They concluded that aggregates height did not change significantly with temperature and the aggregates presented a relative height distribution of 10 nm [66]. There are numerous reports that confirm the conversion of native whey at similar conditions of this report into fibril aggregation, which is an indicator that the aggregates obtained in the present study may be arranged as fibrils [52,63,66,68].

#### 3.2.2. SEM Characterization of Hybrid Membrane of Whey Protein and Activated Carbon

**Figure 2:**
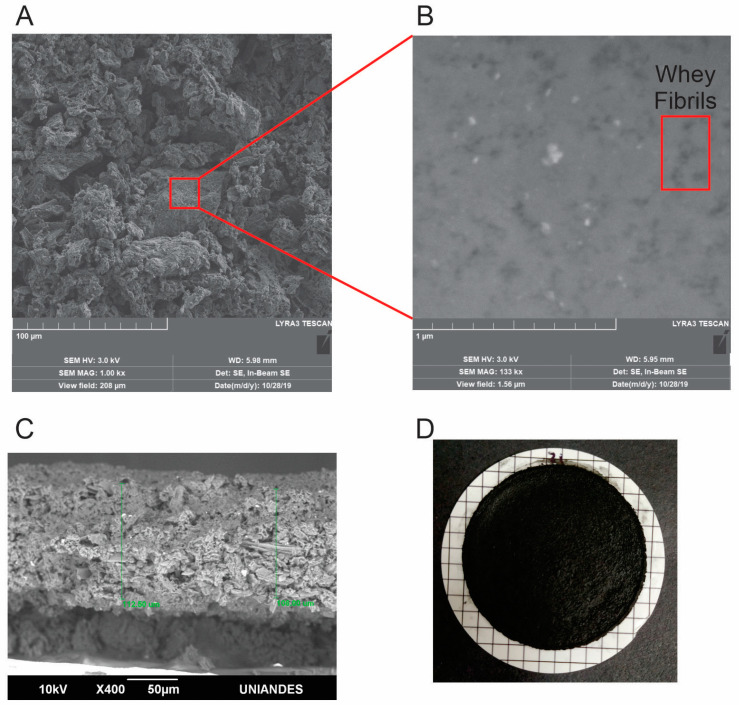
Morphological analysis of hybrid membrane of WPF and AC by SEM. (**A**) Image of the surface of the hybrid nanomembrane; (**B**) higher magnification (100×) of the SEM image of the hybrid membrane, showing in a circle the amyloid WPF adhered to the activated carbon; (**C**) SEM image of the transversal section of the membrane; (**D**) visual aspect of the membrane.

The image of hybrid membrane surface obtained by scanning electron microscopy (SEM) is shown in Figure 2A and the visual aspect of the membrane without magnification is given in Figure 2D, which was prepared with a mixture of 2:1 of WPF and AC by vacuum filtration. To take advantage of the properties of WPF and avoid agglomeration, WPF were adsorbed into AC to increase the mechanical properties and surface area of the WPF to remove heavy metal ions more efficiently.

Figure 2A,C shows the characteristic morphology of activated carbon and the transversal section of the membrane had a thickness of ~ 100 µm ± 10, which is a film thinner than the membrane reported by Peydayesh et al. [18], who prepared a hybrid membrane of β-lactoglobulin amyloid fibrils and activated carbon with a thickness of 1 mm. Consequently, the fact of having a thinner membrane makes that the cross flow of water passes more easily through it. Additionally, the membrane reported in Figure 2D demonstrated to be a rigid film that was sufficiently strong to be handled without disassembling. The same result was found by Li et al. [32] in the preparation of a hybrid membrane of β-lactoglobulin fibrils and graphene oxide showing the robustness of the membrane cutting it with normal scissors [32]. In contrast, hybrid membranes elaborated with just AC demonstrated to be fragile because it disassembled easily to the touch.

Furthermore, Figure 2B shows a higher magnification of the hybrid membrane (1.5 µm), which confirms that amyloid WPF were adsorbed and adhered on the surface of AC thus demonstrating the formation of a uniform hybrid composite. SEM images indicated that WPF were highly adsorbed to the activated carbon surface as β-lactoglobulin fibrils reported by Bolisetty and Mezzenga [16]. In this way, it can be concluded that WPF can be used to elaborate hybrid membranes avoiding the purification of the β-lactoglobulin to obtain high-quality hybrid composites.

#### 3.2.3. Red Congo

Dyeing with congo red (CR) is a method to characterize amyloid fibrillation, which depending on the state of protein aggregation, this dye binds to amyloid fibrils. CR binds to sites parallel and antiparallel to the fibril axis, and antiparallel and parallel, respectively, to the β-sheets on fibrils [34]. To determine the presence of amyloid fibrils by this method, the binding between CR and β-sheet structures is expected to cause an increase in the absorption from approximately 490 (unbound) to 510 nm (bound), with a bathochromic shift in its characteristic absorption spectrum [69].

**Figure 3:**
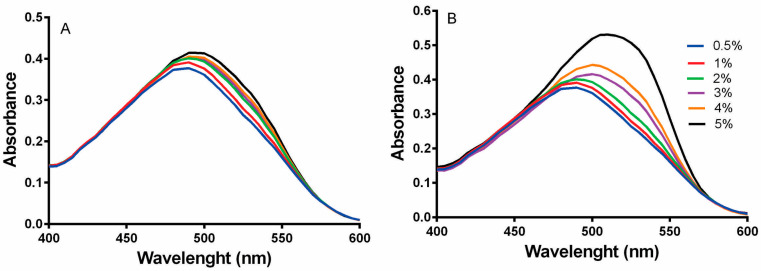
Congo red absorption spectra: (**A**) CR spectra for native whey protein at different concentrations (0.5–5%); (**B**) CR spectra for amyloid WPF at different concentrations after heat treatment. The absorption spectra were recorded from 400 to 600 nm.

The results of Figure 3A show the spectra of native whey protein without heat treatment (as control) at different concentrations (0.5–5%), which had a maximum absorbance at 490 nm. In contrast, Figure 3B shows the whey protein spectra at different concentrations after heat treatment to analyse the amyloid formation. These results demonstrated that concentrations between 0.5 and 2% almost had no effects on the absorbance spectra, but concentrations of 3–4% showed an absorption maximum at 505 nm. Whey protein at 5% showed a significant shift from 490 to 515 nm, showing the formation of maximum amyloid fibrils at this concentration. These changes in absorbance are due to CR gets trapped into the cluster of β-sheet peptide backbone of the aggregates of β-lactoglobulin and α-lactalbumin [70].

These results indicated that a significant change in the absorbance spectra was observed at concentrations higher than 3%, which demonstrated that whey solutions from this concentration after heat treatment aggregated into amyloid fibrils. The same trend was observed by Bolder et al. [39] and Veerman [38] where it has been stated that the increase of conversion of whey into aggregates is due to the increase of the total protein concentrations [38], and that the conversion strongly increases for WPI concentrations exceeding 3% (*w*/*w*) [39]. Furthermore, Nicolai et al. [65] observed that large aggregates were formed a low pH and if a very small fraction of native proteins was depleted and the fraction of aggregates increased becoming more stable with the increase of protein concentration [65]. This method is also a confirmation that the aggregates obtained in this work were arranged as amyloid fibrils.

#### 3.2.4. FTIR

**Figure 4:**
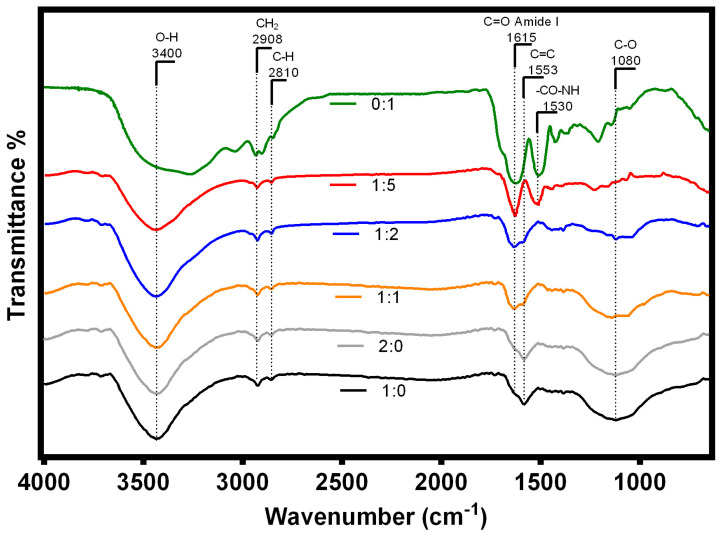
FTIR spectra of hybrid composites with different compositions of activated carbon (AC) and whey fibrils (WF) with respect to whey fibrils alone (green line) and pure activated carbon (black line).

FTIR spectra of hybrid materials at different composition of AC and WPF as well as pure WPF and AC are shown in Figure 4. The results demonstrated that different compositions of WPF and AC exhibited a change on the functional groups of the hybrid composites, which showed characteristic bands of AC and WPF. Consequently, WPF and the hybrid composite with 1:5 of AC and WPF presented a clear intensity variation in the typical amide region between 1300 and 1700 cm^−1^. Note that the band at 1615 cm^−1^ is a characteristic of primary amide group (C=O, C–N) and the band at 1530 cm^−1^ represents the secondary amide group (N–H, C–N) related to peptide bonds (CO–NH) of whey proteins [71]. Additionally, a decrease of intensity or disappearance of the amide bands in the hybrid composites with 1:2, 1:1 and 2:1 of WF and AC was related to the decrease of protein content due to the reduction of the proportion of WPF in these materials.

Moreover, all spectra presented a band at 3400 cm^−1^ that in the case of WPF it corresponds to stretching vibrations of –OH linked to –NH_2_ [72], and it can be also attributed to a hydroxyl group associated to phenols and alcohols for AC and hybrids [73]. All hybrid composites and AC presented bands at 2908 and 2810 cm^−1^ that correspond to C–H aliphatic vibrations, which are assigned to asymmetric CH and symmetric CH bands, respectively, that are related to alkyl groups [74]. Activated carbon and 1:2 hybrid (WPF:AC) spectra showed bands at 3400, 2900, 1550 and 1087 cm^−^^1^ that are related to phenols, alcohols, aliphatic and aromatic rings that are typical bands of activated carbons synthesized at high carbonization time and temperature [75].

The presence of the bands between 1000 and 1380 cm^−1^ in the WPF corresponds to C–O, C–C and C–OH groups [72] that were the ones that became less defined when the content of AC increased in the hybrid composite. Also, the increment of AC content into the hybrid was associated to the band at 1080 cm^−1^ that corresponds to C–O stretching of secondary hydroxyl group, that is well defined in the AC spectra [75]. It may be concluded that WPF were adsorbed into AC where the main changes of hybrid composites spectra in relation with pure WPF and AC were in the region between 900 and 1700 cm^−1^, so that the main changes are related to the functional group of amide for WPF and C–O bonds for AC. A similar behaviour was reported by Torres et al. [76] that described the immobilization of soybean peroxidase into activated carbon where there was a displacement of the band at 1580 cm^−1^ and an overlapping of the C=O band of the backbone of peptide bonds with the N–H, C–N and C=C vibrations.

The results of this study demonstrated that the hybrid composites of WPF and AC presented functional groups of both components suggesting that WPF and AC interacted chemically by amide group and C–O bonds, but also there were physical interactions as hydrophobic forces that include electrostatic, van der Waals, steric and solvation forces carried by amino acids and their polypeptide chain that interacted with AC [77,78]. These findings are consistent with the results presented by Li et al. [32]. These authors identified similar patterns in the FTIR spectrum using composites prepared with amyloid fibrils of β-lactoglobulin and graphene [32]. Consequently, the hybrid composite selected to prepare the hybrid membrane was the hybrid with 1:2 of AC and WPF, which has the band at 1615 cm^−1^ that represents the amide group demonstrating the presence of the peptide bonds of proteins. This means that WPF were adsorbed into AC and its active groups (i.e., –SH, –OH and –NH2 and –COOR of the R group of amino acids) may be present in the hybrid membrane to remove heavy metals. The group –OH and secondary alcohol presented in AC part of the hybrid can also interact with metal ions [79].

#### 3.2.5. BET Surface Area

**Figure 5:**
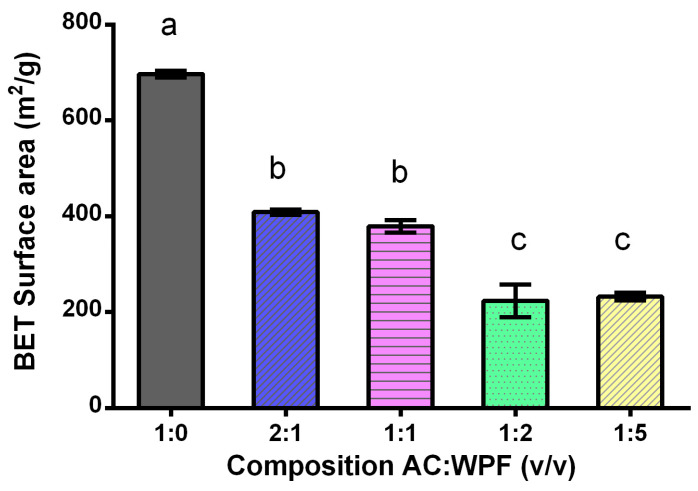
BET surface area of different mixtures of AC and WPF. Error bars are standard deviations (*n* = 3). Different letters on the bars indicate significant differences between different treatments at *p*-level = 0.05 based on the least significant difference (LSD) test.

BET surface area of AC and hybrid composites with different compositions of WPF and AC are shown in Figure 5. The surface area of AC decreased as WPF content in the composite increased, which resulted in an enhancement of the surface area of WPF, thus preventing the agglomeration between them. This is the reason why, at greater quantities of WPF, these adhered to the active empty sites of activated carbon and created a hybrid composite with the features of AC and WPF. Similar findings were observed by Li et al. [32] that characterized a graphene–amyloid fibril (β-lactoglobulin) composite and concluded that amyloid fibrils were adsorbed onto graphene due to charged amyloid fibrils generated an overall surface charge density. They also concluded that when the amount of amyloid fibrils increased, the graphene became more stable in water due to the surface activity of fibrils [32]. Likewise, Andrade et al. [77] studied the adsorption of β-lactoglobulin on AC at pH 3 and they found that the maximum amount of β-lactoglobulin was adsorbed on AC at this pH value. However, the pH of point zero charge of AC was 6 and the isoelectric point of the protein was 5.2 meaning that both were positively charged at these conditions [77]. Additionally, the maximum adsorption of β-lactoglobulin with AC was carried out at pH 3 in accordance with this study, which was carried out at pH 2. In this case, the hydrophobic forces between the amino acids and AC were the driving forces that led to a potential hybrid composite with the properties of WPF and AC, thus allowing the preparation of a hybrid membrane for the removal of heavy metal ions from water [78,80].

Figure 5 shows that the mixtures 2:1 and 1:1 of AC and WPF had a surface area of ~400 m^2^/g, and the maximum WPF adsorbed on AC leaded to a surface area of ~240 m^2^/g using the compositions 1:2 and 1:5 (AC:WPI). From this, it may be concluded that the composition 1:2 was the best mixture to elaborate the hybrid membrane because it was the condition were the AC was saturated with WPF. Therefore, the mixture 1:5 did not have a decrease in its BET area owing to the maximum quantity of fibres adsorbed, leaving fibres in the aqueous medium without being used. The same was observed by Li et al. [32], who identified by TEM a mixture of 1:5 of graphene and β-lactoglobulin fibres left fibre free in the media. Even though, it is well known that materials with a large surface area are good adsorbents, the objective of introducing WPF into AC was to functionalize AC so that the hybrid can compensate the physisorption of the AC with the chemisorption effect of WPF [16]. The hybrid composite from the mixture 1:2 demonstrated to be sufficiently strong to adhere and cover the hole surface of the nitrocellulose membranes by vacuum filtration without disassembling, which was due to the quantity of highly-adherent WPF adsorbed onto AC.

### 3.3. Removal Efficiency of Heavy Metals

**Figure 6:**
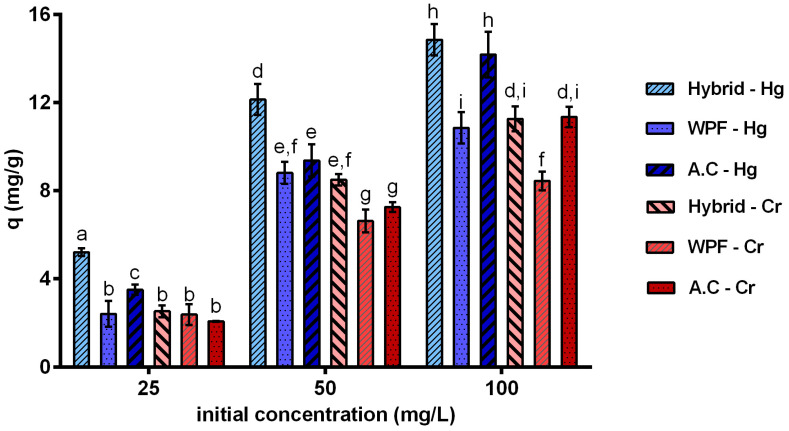
Adsorption capacity of membranes of AC, WPF and the Hybrid of 1:2 AC:WPF for mercury (blue bars) and chromium (red bars) at initial concentrations of 25, 50 and 100 mg/L. Error bars are standard deviations (*n* = 3). Different letters on the bars indicate significant differences between different treatments at *p*-level = 0.05 based on the least significant difference (LSD) test.

The adsorption capacities for batch filtration adsorption of mercury and chromium at 25, 50 and 100 mg/L using a hybrid membrane of WPF and AC are shown in Figure 6. Note that membranes with WPF and AC alone were also analysed as references. As it can be noted, the adsorption capacities for mercury removal were 5.20, 12.14 and 14.85 mg/g at initial Hg concentrations of 25, 50 and 100 mg/L, respectively (Figure 6). The adsorption capacities for the hybrid membrane at 25 and 50 mg/L were significantly different to the results obtained by the membranes of WPF and AC alone. This could be due to the synergistic effect of the AC and the WPF because both materials created a balance between the porosity of the membrane and the operating water processing flow rate [16]. It can explain that the membranes of WPF alone showed agglomerations of fibrils that were fixed on the surface of the cellulose membrane by the hydrophobic character of WPF and it did not allow an efficient mass transport on the membrane.

The adsorption capacity of the hybrid membrane was 14.85 mg/g for mercury at initial concentration of 100 mg/L, which was a higher value regarding to the adsorption capacity of 10.61 and 9.61 mg/g reported by Bolisetty and Mezzenga [16] using a hybrid membrane made of β-Lactoglobulin and a hybrid membrane of melanin-coated with PVDF, respectively, see Table 4. However, Razmi et al. [81] obtained an adsorption capacity of 77 mg/g to remove mercury using a hybrid membrane of eggshell protein with reduced graphene oxide, which presented a higher adsorption capacity due to the functional groups, high surface volume ratio and uniform distribution of the reduced graphene oxide [81]. From this analysis, it can be inferred that the hybrid membrane of whey protein fibrils is even more efficient as one made of just pure β-Lactoglobulin, but it can also be improved by changing the support of the WPF by reduced graphene oxide or using other types of adsorbent.

Similarly, the results of the adsorption capacities for the hybrid membrane were 2.53, 8.5 and 11.26 mg/g to remove chromium at initial concentrations of 25, 50 and 100 mg/L. There was no significant evidence to claim that the adsorption capacities of the hybrid membrane were different from the membranes with WPF and AC alone. Yet, the hybrid membrane showed adsorption results as good as the results of a membrane made with commercial activated carbon alone, which demonstrated the high potential of this hybrid material for heavy metal removal. Note that Peydayesh et al. [18] reported an adsorption capacity for β-lactoglobulin of 5.7 times higher than activated carbon for chromium at 174 ppb [18]. Additionally, the hybrid membrane of WPF and AC showed better removal for Cr than the hybrid membrane of melanin nanoparticles coated in polyvinylidene fluoride membrane obtained by Manirethan and Balakrishnan [84] (see Table 4). However, some of the bio-based hybrid membranes reported in the literature showed a better performance to remove Cr, comparison to the hybrid membrane of this study, with adsorption capacities of 126.7 and 148.65 mg/g using a hybrid membrane of silk nanofibrils and hydroxyapatite [83] and a hybrid membrane of AC and β-lactoglobulin [18] at tested conditions. The high adsorption capacity of the hybrid membrane showed a superior performance compared with the results of this study due to silk nanofibrils and hydroxyapatite interact with metal ions through chelation and ion exchange, besides the favourable conditions of pH [83]. Furthermore, it is important to remark that the result of the adsorption capacity of the hybrid membrane of AC and β-lactoglobulin [18] with respect to the result obtained in this study are not comparable as the initial concentrations evaluated differ by 1000 times. However, the higher performance of the hybrid membrane of AC and β-lactoglobulin could be due to the active functional groups of the isolated β-lactoglobulin [18]. In contrast, the adsorption capacities obtained in this study for mercury were higher than the adsorption capacities for chromium, which agreed with the results for WPF, which appears to have more affinity for the removal of mercury than chromium. These results confirmed the prominent role of WPF in the separation performance of these hybrid membranes [17]. Consequently, it opens the possibility to use WPF alone to elaborate hybrid membranes [56] to remove mercury, chromium and other heavy metals as is shown by other reports, based on the fact that some of the functional groups that work to remove Hg well may also work to remove other heavy metals, such as Pb [82], Pd [16] and As [17]. Additionally, it is possible to design other types of hybrid membranes using WPF to avoid the use of commercial AC [46].

**Figure 7:**
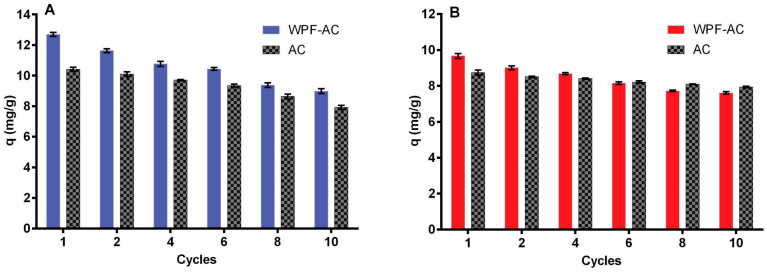
Comparison of the adsorption capacity per filtration cycle of the hybrid membrane and AC alone using adsorbate solutions with initial concentration of 50 mg/L. (**A**) Adsorption capacity per cycle of filtration for mercury; (**B**) adsorption capacity per cycle of filtration for chromium. Error bars represent the standard deviations (*n* = 3).

The reusability of the hybrid membrane is shown in Figure 7 where 50 mL of a solution of HgCl_2_ (Figure 7A) and Na_2_CrO_4_ (Figure 7B) with an initial concentration of 50 mg/L were filtered by ten consecutive cycles with the same hybrid membrane. The results showed that the performance of the membrane was efficient beyond the ten cycles, but the adsorption capacities decreased as the number of filtering cycles increased. Note that these decrements of the removal performance were more significant for the hybrid membrane in comparison with the results obtained with an AC membrane. This phenomenon could be due to the impact of mass transfer phenomena on the adsorption mechanism due to textural parameters [85]. Reduction of metal adsorption could be the consequence of the decrease in 65% of the BET area of the hybrid material with respect to the AC membrane. Considering that AC has a large surface area due to the high number of micropores [86], the saturation of saturation of AC membrane requires more cycles than the hybrid material. However, the mercury removal of the hybrid membrane was still higher than the AC membrane due to the chemisorption effects and electrostatic interactions of the functional groups with high affinity for Hg [47]. Similar findings were reported by Bolisetty and Mezzenga [16]. These authors identified a high saturation capacity for a hybrid membrane of β-lactoglobulin through 10 filtering cycles for KAu(CN)_2_, HgCl_2_, Pb(C_2_H_3_O_2_)_4_ and Na_2_PdCl_4_. Bolisetty et al. [17] stated that this type of membrane can be used for several cycles without its considerable saturation for arsenic removal. The same effect was also noted by different studies on the removal of chromium, nickel, silver, platinum and aluminum [2,18].

## 4. Conclusions

In this work, whey protein fibrils were produced by heat treatment and used to remove chromium and mercury from water. Results showed that the best conditions to produce WPF were 74 °C, 7 h and 3.8% of whey protein, which demonstrated a better adsorption capacity for mercury than chromium in a mixed solution and alone. Removal studies confirmed the WPF affinity for mercury due to WPF having more active groups that interact with mercury in contrast with the electrostatic interactions between the positive charge of WPF and the negative charge of HCrO_4_^2−^. It has been demonstrated that the operational conditions to produce WPF as temperature, time and whey content have a critical effect on whey protein aggregation and, consequently, on the adsorption capacity of heavy metals. Consequently, WPF in combination with AC were used to produce a hybrid membrane, which, as confirmed by SEM, resulted in a high-quality, rigid and well-organized layered structures. FTIR and Red Congo assays led to the conclusion that the hybrid membrane obtained have the functional groups of AC and WPF and that the whey proteins of the membrane are arranged as amyloid fibrils, respectively. The hybrid membrane of WPF and AC efficiently removed mercury and chromium from water with adsorption capacities of 14.85 and 11.26 mg/g using batch vacuum filtration, supporting beyond ten filtration cycles. The performance of the removal of mercury and chromium is due to the synergistic effect of the chemisorption of the attracting functional groups of WPF by heavy metals and the physisorption of AC pores, which led to a hybrid membrane suitable for the removal of Hg and Cr. The promissory performance of this hybrid membrane leads to the possibility of using the mixture of proteins like whey to produce fibre amyloids as a way to avoid the separation of β-lactoglobulin or other proteins from whey, which results in a lower cost production, and a convenient and quicker process. Furthermore, it is a way to give an application to whey that is a cheese byproduct.

## Figures and Tables

**Table 1:** Description of the central composite design (CCD) used for WPF preparation and its impact on mercury and chromium removal from water.

Variable.	Unit	Factors	Level				
			−α	−1	0	1	α
Temperature	°C	A	66	70	80	90	94
Time	h	B	5	6	8	10	11
Whey concentration	wt%	C	0.4	1	2.5	4	4.6

α = 1.41 (star or axial point for orthogonal small CCD in the case of three independent variables and their actual values were rounded).

**Table 2:** Results of Central Composite Design (CCD) for WPF preparation and its impact on mercury and chromium removal from water, experimental and analytical response. Factors: A—Temperature (°C), B—time (h) and C—whey concentration (wt%).

Run	A	B	C	Adsorption Capacity and Efficiency for Hg						Adsorption Capacity and Efficiency for Cr					
				Experimental		Predicted		Error		Experimental		Predicted		Error	
				q	%	q	%	q	%	q	%	q	%	q	%
1	66	8	2.5	23.4	79	23.5	79	0.5	0.5	17.8	56	17.9	56	0.6	0.6
2	70	6	1	13.9	47	13.8	46	0.9	0.9	11.4	36	11.3	35	1.1	1.1
3	70	10	4	12.6	42	12.4	42	1.1	1.0	9.5	30	9.3	29	1.4	1.4
4	80	5	2.5	23.0	78	23.1	78	0.5	0.5	9.8	31	9.9	31	1.1	1.1
5	80	8	0.4	2.6	8	2.7	9	4.7	4.7	1.5	4	1.6	5	7.5	7.6
6	80	8	2.5	21.5	73	20.1	68	6.9	6.9	7.5	23	7.7	24	2.5	2.5
7	80	8	2.5	20.0	68	20.1	68	0.4	0.4	8.0	25	7.7	24	3.7	3.7
8	80	8	2.5	20.2	68	20.1	68	0.7	0.7	7.4	23	7.7	24	4.0	4.0
9	80	8	2.5	18.9	64	20.1	68	6.1	6.1	8.0	25	7.7	24	3.0	3.0
10	80	8	2.5	20.1	68	20.1	68	0.2	0.3	7.9	25	7.7	24	2.2	2.2
11	80	8	4.6	15.5	52	15.6	53	0.6	0.7	17.2	54	17.3	54	0.6	0.6
12	80	11	2.5	13.0	44	13.1	44	0.8	0.9	6.6	21	6.8	21	1.6	1.6
13	90	6	4	6.5	21	6.3	21	2.1	2.1	10.7	33	10.6	33	1.2	1.2
14	90	10	1	24.2	82	24.1	81	0.6	0.5	6.6	21	6.5	20	2.0	2.0
15	94	8	2.5	17.4	59	17.5	59	0.6	0.7	2.1	6	2.2	7	4.9	5.0

**Table 3:** Best preparation conditions to produce WPF and to improve the adsorption capacity and removal efficiency for mercury and chromium. Validated values were expressed as mean ± SD of triplicate.

Best Preparation Conditions		
	Mercury	Chromium
Temperature (°C)	74.1	73.5
Time (h)	7.5	7.1
Protein concentration (%)	3.8	3.7
q (mg/g) predicted	29.4	19.3
q (mg/g) validated	24.9 ± 0.3	17.9 ± 0.8
q (mg/g) mixed	24.1 ± 0.8	11.5 ± 0.8
q (mg/g) β-Lactoglobulin	27.7± 0.2	19.35 ± 1.3
Error (%)	15.2	6.8
Removal efficiency (%) predicted	100	61
Removal efficiency (%) validated	81 ± 0.5	57 ± 1.8
Removal efficiency (%) Mixed	78 ± 1.4	37 ± 1.6
Removal efficiency (%) β-Lactoglobulin	90 ± 1.2	62 ± 2.1
Error (%)	19	5

**Table 4:** Metal adsorption capacity of some bio-based hybrid membranes reported in literature.

Hybrid Membrane	Heavy Metal	q (mg/g)	Evaluation	Co (mg/g)	Ref
Hybrid membrane AC-B-lactoblogulin	Au	52.5	1st Cycle	561	[16]
	Hg	10.6		85	
	Pb	995.7		65	
	Pd	356.6		12	
Eggshell membrane protein doped with reduced graphene oxide	Hg	77	1st Cycle	0.01	[81]
Hybrid membrane AC-B-lactoblogulin	Arsenate	0.2	1st Cycle	0.2	[17]
	Arsenite	1.1			
Hybrid membrane of lignin/nylon-6 membrane and oats/nylon-6 membrane	Pb	37 and 11	Batch for 2h	10	[82]
Hybrid membrane of silk nanofibril (SNF) and hydroxyapatite (HAP)	Au	135.7	Batch for 48 h	100	[83]
	Ag	-			
	Cu	64.7			
	Ni	63.0			
	Cr	126.7			
	Pb	-			
Hybrid membrane AC-B-lactoblogulin	Cr	148.6	1st Cycle	0.17	[18]
	Ni	13.8		0.21	
	Ag	86.7		0.20	
	Pt	234.7		0.13	
Graphene–Bovine Serum Albumin Hybrid Membrane	Co	0.5	1st Cycle	1000	[2]
	Cu	0.3			
Melanin-coated PVDF membranes	Hg	9.2	1st Cycle	3	[84]
	Cr	5.0			
	Pb	4.0			
	Cu	6.9			
This study	Hg	14.8	1st Cycle	100	
	Cr	11.2			
